# Poster 2004: Bio Immun(G)En medicine or how to link scientific research and daily therapeutic practice

**DOI:** 10.1186/1939-4551-7-S1-P20

**Published:** 2014-02-03

**Authors:** Gilbert Glady

**Affiliations:** 1 EBMA, Colmar, France

## Background

The main object of our particular approach in **Bio Immune(G)enetic Medicine**, is to use the reaction models specific to the human immune system, for the purpose of not only diagnosis but also - and more especially –therapy. In this respect it resembles theranostic nanomedicine.

## Methods

To reach diagnosis we use a wide range of biological parameters such as lymphocyte typing and protein profiles as well as many bacterial and viral serologies, but also biomarkers such as thymidine-kinase or transketolase-1. Indeed, taken together, these factors enable us, on the one hand to substantiate a precise and relevant biological diagnosis, and on the other to obtain a guide for suitable subsequent therapy.

The therapy uses numerous immune-competent molecules, in particular microRNAs, which are involved in one of the principal epigenetic processes currently known. They are prepared according to the homeopathic dilution-dynamisation mode, this being the sole means of ensuring total safety in terms of any adverse side-effects for the patient, and therapeutic effectiveness is based on the universal today well-known principle of Hormesis.

## Results

This approach enables the development of a bio-mimetic immune therapy, the medium-to-long-term regulatory effects of which, are proving to be extremely beneficial to patients in a large number of immuno-dependent diseases.

## Conclusions

The description of various clinical cases will demonstrate this innovative method and provide a clear example of how discoveries in the field of basic research can be applied directly in the domain of clinical and therapeutic medicine by humans.

